# Detection and host associations of Nairobi sheep disease virus in the human upper respiratory tract in Beijing, China

**DOI:** 10.3389/fmicb.2026.1849489

**Published:** 2026-07-01

**Authors:** Liu Pengfei, Lin Chenyu, Hou Runyu, Xie Lixin

**Affiliations:** 1 College of Pulmonary and Critical Care Medicine, The 8th Medical Centre of Chinese PLA General Hospital, Beijing, China; 2 Nanhu Laboratory, State Key Laboratory of Biomedical Analysis (SKLBA, Formerly Known as National Center of Biomedical Analysis, NCBA), Beijing, China; 3 Emergency Medicine, The 8th Medical Centre of Chinese PLA General Hospital, Beijing, China

**Keywords:** fever, infection immunity, Nairobi sheep disease virus, tick-borne virus, virus-host association analysis

## Abstract

**Objective:**

To address the serious threat posed by emerging viral pathogens in the human upper respiratory tract against the backdrop of global epidemics of various viruses, particularly the coronavirus disease 2019 (COVID-19) pandemic in recent years, this study aimed to identify previously undetected in the human upper respiratory tract viruses in febrile patients, clarify their clinical and epidemiological characteristics, and analyze virus-host interactions through multi-omics approaches, thereby providing a scientific basis for the prevention and treatment of newly discovered upper respiratory tract viruses.

**Methods:**

This was a single-center cross-sectional study conducted at the fever clinic of the Eighth Medical Center of the People’s Liberation Army (PLA) General Hospital in Beijing, China, from April 2025 to May 2025. Two years after the outbreak of severe acute respiratory syndrome coronavirus 2 (SARS-CoV-2), throat swab samples were collected from 218 febrile patients. Next-generation sequencing (NGS) was performed, and the obtained sequences were comprehensively analyzed using the Global Pathogen Analysis System (GPAS). Samples that tested positive for Nairobi sheep disease virus (NSDV) were further subjected to serum antigen verification, followed by serological antibody follow-up at 4 months post-diagnosis. Additionally, multi-omics approaches were employed, along with intergroup comparisons of multicategorical ordinal variables and logistic regression analysis, to assess virus-host correlations.

**Results:**

Among the 218 patients, NSDV, a tick-borne virus, was detected in the throat swab samples of 10 patients, of whom 2 remained seropositive for NSDV antibodies 4 months after diagnosis. Further correlation analysis among NSDV positive patients revealed that tumor was the most significant and statistically meaningful risk factor for NSDV positivity, with an odds ratio (OR) of 3.330 (95% confidence interval [CI]: 1.352–8.199; *p* = 0.0089). Increased age was associated with a decreased risk of NSDV positivity (OR = 0.949; 95% CI: 0.913–0.987; *p* = 0.0085). Smoking and positive imaging findings may constitute potential risk factors.

**Conclusion:**

NSDV can be detected in the human upper respiratory tract and may contribute to febrile illness in the post-pandemic era. Persistent seropositivity suggests possible long-term immune engagement, and multi-omics analyses help further elucidate host susceptibility and viral adaptation mechanisms.

## 1 Introduction

The coronavirus disease 2019 (COVID-19) pandemic has caused millions of deaths worldwide since the beginning of 2020 (Ramasamy and Subbian, 2021). In addition to severe acute respiratory syndrome coronavirus 2 (SARS-CoV-2), many other viruses and related infectious diseases have posed a serious threat to public health, and influenza viruses are frequently detected among various respiratory pathogens. The outbreak of seasonal influenza causes about 250,000–500,000 global deaths each year (Zhang et al., 2021). Given the many risks posed by numerous emerging viruses, it is essential to explore the relationship between emerging viruses and their hosts, especially the antagonistic relationship between viruses and host antiviral innate immunity (Lum et al., 2022). Accelerating the research progress of the antagonism of emerging viruses to host antiviral innate immunity may provide a more comprehensive insight into emerging viruses and their pathogenic mechanisms, which contributes to the rapid response to the outbreak of mutant strains and global epidemics.

In this study, a tick-borne virus, Nairobi sheep disease virus (NSDV), which latently infected the human upper respiratory tract, was identified. NSDV was first isolated from the blood of affected sheep in 1910. NSDV infection in humans can cause febrile diseases manifested as abdominal and back pain, headache, nausea, and vomiting (Abudurexiti et al., 2019; Goldsmith et al., 2013). Due to the presence of animal hosts, these diseases are prevalent in nature. When humans enter natural epidemic foci, they may be infected with these viruses. Currently, NSDV-specific antibodies have been isolated from ticks, humans, sheep, and mosquitoes in India and Africa, and NSDV-positive antibodies have also been reported in the serum of humans, sheep, goats, and cattle (Liu et al., 2014).

In China, NSDV was first detected by the reverse transcription-polymerase chain reaction (RT-PCR) in *Haemaphysalis longicornis* in Northeast China as early as 2015. However, NSDV infections or disease outbreaks in goats or sheep have not been reported in China. In 2019, the serological evidence of NSDV infection in *Haemaphysalis longicornis* and goats in Hubei province (China) was reported. Specifically, non-infectious viruses were found in the ticks from goats, but the non-infectious NSDV-positive serum was not found in these goats (Yang et al., 2019).

It is worth noting that there have been no reports on the detection of this virus in the respiratory tract and serum of the Chinese population. Furthermore, the detection results of NSDV antibodies in human serum and the epidemiological investigation findings related to this virus were also elucidated in this study, and analyze virus-host interactions through multi-omics approaches, thereby providing a scientific basis for the prevention and treatment of newly discovered upper respiratory tract viruses.

## 2 Methods

### 2.1 Inclusion and exclusion criteria and ethical statement

This study is a single-center, cross-sectional investigation designed to detect latent tick-borne viruses in the upper respiratory tract of febrile patients in Beijing using metagenomic Next-generation sequencing (mNGS) and Global Pathogen Analysis System (GPAS) analysis. In April 2025, 1 year after the outbreak of SARS-CoV-2, influenza viruses became prevalent in Beijing (China), leading to post-infection fever in a wave of patients. A total of 218 patients who were diagnosed with post-infection fever in the fever clinic of the 8th Medical Centre of Chinese People’s Liberation Army (PLA) General Hospital from April 2025 to May 2025 were included in this study.

The inclusion criteria included: (1) patients (aged 18–85 years) who were preliminarily diagnosed with respiratory viral infections; (2) patients with a body temperature above 37.3 °C within the past 5 days; (3) patients with at least one of the following symptoms of upper respiratory tract infection, including nasal congestion, runny nose, sneezing, coughing, expectoration, and pharyngalgia; (4) patients with respiratory tract infection who could undergo throat swab collection and consented to submit samples for pathogen detection. The exclusion criteria included: (1) patients who did not agree to be included in this study or had no samples for pathogen detection; (2) patients who were admitted to the hospital due to the acute exacerbation of such diseases as organ failure, advanced cancer, acute pulmonary embolism, and acute coronary syndrome that may seriously affect their lives. In contrast, 175 healthy controls who underwent throat swab collection in the health examination center of the 8th Medical Centre of Chinese PLA General Hospital within the same period were also included in this study.

This study was performed in accordance with the ethical standards of the Declaration of Helsinki. All subjects understood the purpose and content of the study and provided signed informed consent. This study was approved by the Ethics Committee of Chinese PLA General Hospital (No. 30920240422503033).

### 2.2 Sampling, DNA extraction, and metagenomic analysis

The standardized throat swab samples were collected from these 218 patients and 175 healthy controls. Specifically, a throat swab was used to wipe the bilateral pharyngeal tonsil (three times for the upper and lower sides) and the posterior pharyngeal wall (three times for the upper and lower sides and the left and right sides, respectively) of these subjects to collect samples. Then, these samples were cryopreserved at 4 °C and transported via the cold chain to the testing facility. The duration between admission, sampling, and testing was controlled within 10 h. These samples were subjected to NGS according to existing protocols.

DNA was extracted from 1mL BALF samples using a QIAamp® UCP Pathogen DNA Kit (Qiagen, Valencia, CA, USA) according to the manufacturer’s instructions. Human DNA was removed using Benzonase (Qiagen, Valencia, CA, USA) and Tween20 (Sigma, St. Louis, Missouri, USA). The QIAamp® Viral RNA Kit (Qiagen, Valencia, CA, USA) was applied to extract total RNA, and ribosomal RNA was removed using a Ribo-Zero rRNA Removal Kit (Illumina, San Diego, CA, USA). RNA was reverse transcribed, and amplified by Ovation RNA-Seq system (NuGEN, CA, USA). Following fragmentation, the library was constructed based on combined DNA and reverse transcribed using Ovation Ultralow System V2 (NuGEN, CA, USA) and the concentration of the library was measured using Qubit. Sequencing was executed on Illumina Nextseq 550Dx with 75-bp single-end reads. For negative controls, peripheral blood mononuclear cell (PBMC) samples with 10^5^ cells/mL from healthy donors in parallel were prepared with each batch, using the same protocol, and sterile deionized water was extracted alongside the samples to serve as non-template controls (NTC). NTCs were subjected to the complete assay workflow, mirroring the procedures applied to clinical samples, commencing with nucleic acid extraction.

Raw sequencing data was processed using software Fastp to remove reads containing adapters or ambiguous “*N*” nucleotides and low-quality reads. Low-complexity reads were removed by Kcomplexity with default parameters. Human sequence data were identified and excluded by mapping to a human reference genome (hg38) using Burrows–Wheeler Aligner software. Microbial reads were then aligned to self-building database with SNAP v1.0 beta.18. Approximately 20 million reads were generated for each sample. For pathogen with background reads in negative control, a positive detection was reported for a given species or genus if the reads per million (RPM) ratio was ≥10, where the RPM ratio was defined as the RPMsample/ RPMNTC. For pathogen without background reads in negative control, the RPM threshold for a positive detection was set ≥0.05.

For the analysis of sequencing data, initial preprocessing of raw data involved the removal of low-quality sequences, residual adapters, and reads of insufficient length. Sequences corresponding to microbial rRNA and human genomic material were also excluded. The refined data set was then aligned and annotated against a self-building database of pathogenic microorganisms, leveraging BLAST software for sequence comparison. Non-duplication reads aligning with the target capture regions were classified as target reads and normalized to RPM to facilitate quantitative comparisons.

The sequences obtained in NGS were analyzed thoroughly using the global pathogen analysis system (GPAS).^1^ Additionally, the general information of these subjects was also collected, including age, gender, body temperature, clinical symptoms, and related physicochemical examination data. Besides, the medical history of these subjects was also recorded. The informed consent for data collection was obtained from these subjects. Among these 218 patients, a tick-borne virus (namely NSDV), which was not identified in the Chinese population in the previous, was detected in the throat swab samples of 10 patients. Epidemiological investigation and serum antigen verification were carried out for 5 patients with NSDV infection (The other five individuals declined to revisit the hospital and provide blood samples for research testing).

The throat swabs and sampling transport tubes described in the article are both supplied by Shenzhen Medico Biomedical. The throat swab is a sterile, single-use sampling swab, model MFS-98000KQ-B. The sampling and transport tube is made of medical-grade polypropylene (PP), model MVTM-10B (MH). The inactivating preservation solution within the tube contains highly efficient viral lysis agents, such as guanidinium salts (guanidine isothiocyanate, guanidine hydrochloride, etc.), which rapidly lyse viral proteins and inactivate the virus, substantially reducing the risk of secondary infection for healthcare and transport personnel. Furthermore, an RNase inhibitor added to the preservation solution prevents nucleic acid degradation, ensuring that the viral nucleic acid remains stable during storage and transport at ambient temperature for a defined period, thereby facilitating subsequent PCR testing. For further details, please refer to the official website: https://www.medical-swab.com.

### 2.3 Environment investigation and hospital monitoring

In this study, 5 patients with NSDV infection were investigated from the epidemiological perspective, focusing on the vegetation cover of their living and working environments within 4 months before and after the detection of NSDV. In addition, their clinical data on the medical treatment in the 8th Medical Centre of Chinese PLA General Hospital within 4 months before and after the detection of NSDV were also analyzed.

### 2.4 Serological detection

The antibody levels against the NSDV Gn protein in serum were detected using an indirect enzyme-linked immunosorbent assay (ELISA). The brief procedure was as follows: Recombinant expressed and purified His-tagged NSDV Gn antigen (1 μg/mL, dissolved in carbonate coating buffer) was coated onto a 96-well microplate at 100 μL per well and incubated overnight at 4 °C. After washing, the plates were blocked with PBST containing 5% skim milk at room temperature for 2 h. Subsequently, the test serum samples and control samples, diluted 1:100, were added to the corresponding wells and incubated at 37 °C for 1 h. After washing, horseradish peroxidase (HRP)-labeled goat anti-human IgG secondary antibody (diluted 1:5000) was added and incubated at 37 °C for 1 h. Following another wash, TMB substrate solution was added for color development, and the reaction was incubated at room temperature protected from light for 15 min. Finally, the reaction was terminated with 2 M H₂SO₄, and the absorbance was immediately measured at a wavelength of 450 nm. The following controls were included in the experiment: serum from two confirmed healthy individuals served as the negative control; the coated His-tagged antigen served as the positive control; and a blank control (only substrate and stop solution added) was also set up.

### 2.5 Statistical analysis

Excel was used for data entry and collation. Statistical analysis was performed using SAS 9.4.

The statistical analysis was performed based on (1) baseline indicators: age and gender; (2) clinical symptoms; (3) laboratory examination indicators: white blood cells, neutrophils, lymphocytes, and C-reactive protein (CRP).

The quantitative indicators were described as the number of cases (N), mean (Mean), standard deviation (SD), median (Median), quartile (Q1, Q3), minimum value (Min), and maximum value (Max); while the qualitative indicators were described as the number of cases, rate, or constituent ratio. The inter-group comparison of quantitative measures was performed using either the *t*-test or Wilcoxon rank-sum test; while the intra-group comparison was performed using the paired *t*-test or Wilcoxon signed-rank test. The inter-group comparison of bicategorical variables and multi-categorical nominal variables was performed using the χ^2^ test or Fisher’s exact probability test; while the inter-group comparison of multi-categorical ordinal variables was performed using the Wilcoxon rank-sum test. Logistic regression was used for the multivariate analysis of immune evaluation indicators. The Hosmer-Lemeshow test was conducted to evaluate the goodness of fit of the models. The test level was defined as α = 0.05.

## 3 Results

### 3.1 NSDV characterization

NSDV has a negative-sense single-stranded trilateral RNA genome that can encode L protein, glycoprotein precursor, and N protein. The L, M, and S fragments have typical terminal reverse complement sequences, comprising the 5′ end of UCUCAAAGA and the 3′ end of AGAGUUUCU.

### 3.2 NSDV infection in the upper respiratory tract of patients

**Figure 1:**
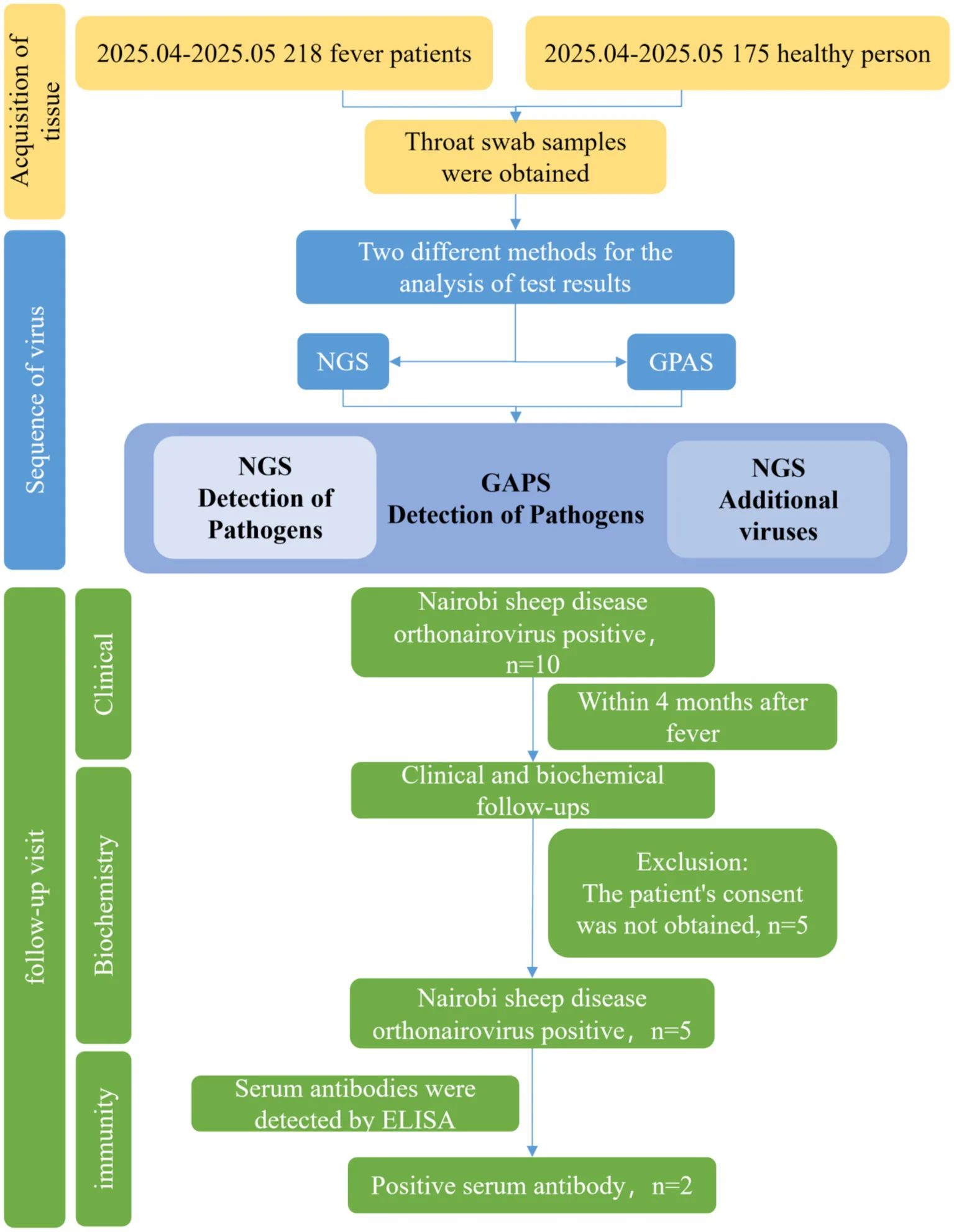
Flow chart of study design.

In this study, the analysis based on metagenomic next-generation sequencing (mNGS) and GPAS was performed on 218 patients and 175 healthy controls. Besides, other viruses detected by GPAS were further explored. The study protocol is illustrated in Figure 1. The median age of these patients was 35.5 years (range: 18–80 years), and female patients accounted for 45.9%. In addition to fever, these patients also presented with nasal congestion (52.3%), sneezing (54.1%), runny nose (33.0%), coughing (70%), expectoration (52.3%), sore throat (69.3%), muscle soreness (76.1%), and fatigue (69.7%). The laboratory examination results indicated that there were significant differences in the absolute lymphocyte count (2.38 ± 13.17; 1.22 ± 4.31; *p* = 0.0054) and C-reactive protein level (16.11 ± 15.72; 10.23 ± 5.56; *p* = 0.0026) between these patients and healthy controls, which was consistent with the hemogram results of viral infection in clinical practice (Table 1).

**Table 1:** Information on the studied populations in this study.

Epidemiology character	Fever patients (*n* = 218; %)	Health comparison (*n* = 175; %)	The NSDV-positive patients were detected by GPAS (*n* = 10; %)	The NSDV-positive patients were detected by Elisa (*n* = 2; %)
Age (18–80)				
<40	130 (59.6)	48 (27.4)	8 (80)	2 (100)
40–60	61 (28.0)	95 (54.3)	2 (20)	0 (0)
>60	27 (12.4)	32 (18.3)	0 (0)	0 (0)
Mean (SD)	39.06 (15.25)	43.63 (12.34)		
Median (Q_1_, Q_3_)	35.50 (27.00, 48.25)	44 (29.00, 54.00)		
Min, Max	18, 80	21, 80		
Statistic	0.54			
*P*	0.4035			
Sex				
Male	118 (54.1)	105 (60.00)	5 (50.0)	1 (50.0)
Female	100 (45.9)	70 (40.00)	5 (50.0)	1 (50.0)
Statistic	0.05			
*P*	0.8277			
Clinical manifestation				
Stuffed nose	114 (52.3)		4 (40.0)	1 (50.0)
Running nose	118 (54.1)		5 (50.0)	1 (50.0)
Sternutation	72 (33.0)		3 (30.0)	0 (0)
Cough	157 (70.0)		7 (70.0)	2 (100)
Sputum	114 (52.3)		7 (70.0)	2 (100)
Sore throat	151 (69.3)		7 (70.0)	1 (50.0)
Muscular stiffness	166 (76.1)		9 (90.0)	1 (50.0)
Malaise	152 (69.7)		9 (90.0)	1 (50.0)
White blood cell (10^9^/L)				
Low (<3.5)	11 (5.0)	17 (9.7)	1 (10.0)	0 (0)
*N* (3.5–9.5)	188 (86.2)	153 (87.4)	9 (90.0)	2 (100)
High (>9.5)	19 (8.7)	5 (2.9)	0 (0)	0 (0)
Mean (SD)	6.68 (2.00)	6.77 (3.83)		
Median (Q_1_, Q_3_)	6.58 (5.26, 8.46)	5.76 (2.16, 6.21)		
Min, Max	0.53, 10.94	0.76, 26.96		
Statistic	0.06			
*P*	0.9535			
Neutrophil (10^9^/L)				
Low (<2.0)	17 (7.8)	9 (5.1)	2 (20.0)	0 (0)
*N* (2.0–7.5)	192 (88.1)	151 (86.3)	8 (80.0)	2 (100)
High (>7.5)	9 (4.1)	15 (8.6)	0 (0)	0 (0)
Mean (SD)	4.52 (1.85)	4.86 (3.83)		
Median (Q_1_, Q_3_)	4.41 (3.17, 6.12)	3.67 (2.16, 6.21)		
Min, Max	0.14, 9.12	0.43, 9.21		
Statistic	0.23			
*P*	0.8170			
Lymphocyte (10^9^/L)				
Low (<1.1)	99 (45.4)	17 (9.7)	7 (70.0)	1 (50)
*N* (1.1–3.2)	110 (50.5)	147 (84)	3 (30.0)	1 (50)
High (>3.2)	9 (4.1)	11 (6.3)	0 (0)	0 (0)
Mean (SD)	2.38 (13.17)	1.22 (4.31)		
Median (Q_1_, Q_3_)	1.20 (0.79, 1.79)	1.04 (0.62, 1.50)		
Min, Max	0.01, 193.00	0.16, 14.52		
Statistic	2.78			
*P*	0.0054			
C-reactive protein (mg/L)				
*N* (0–10)	101 (46.3)	145 (82.9)	7 (70.0)	1 (50)
High (>10)	117 (53.7)	30 (17.1)	3 (30.0)	1 (50)
Mean (SD)	16.11 (15.72)	10.23 (5.56)		
Median (Q_1_, Q_3_)	11.26 (5.14, 23)	8.97 (4.40, 18.73)		
Min, Max	0.00, 95.65	0.00, 41.68		
Statistic	3.01			
*P*	0.0026			

**Figure 2:**
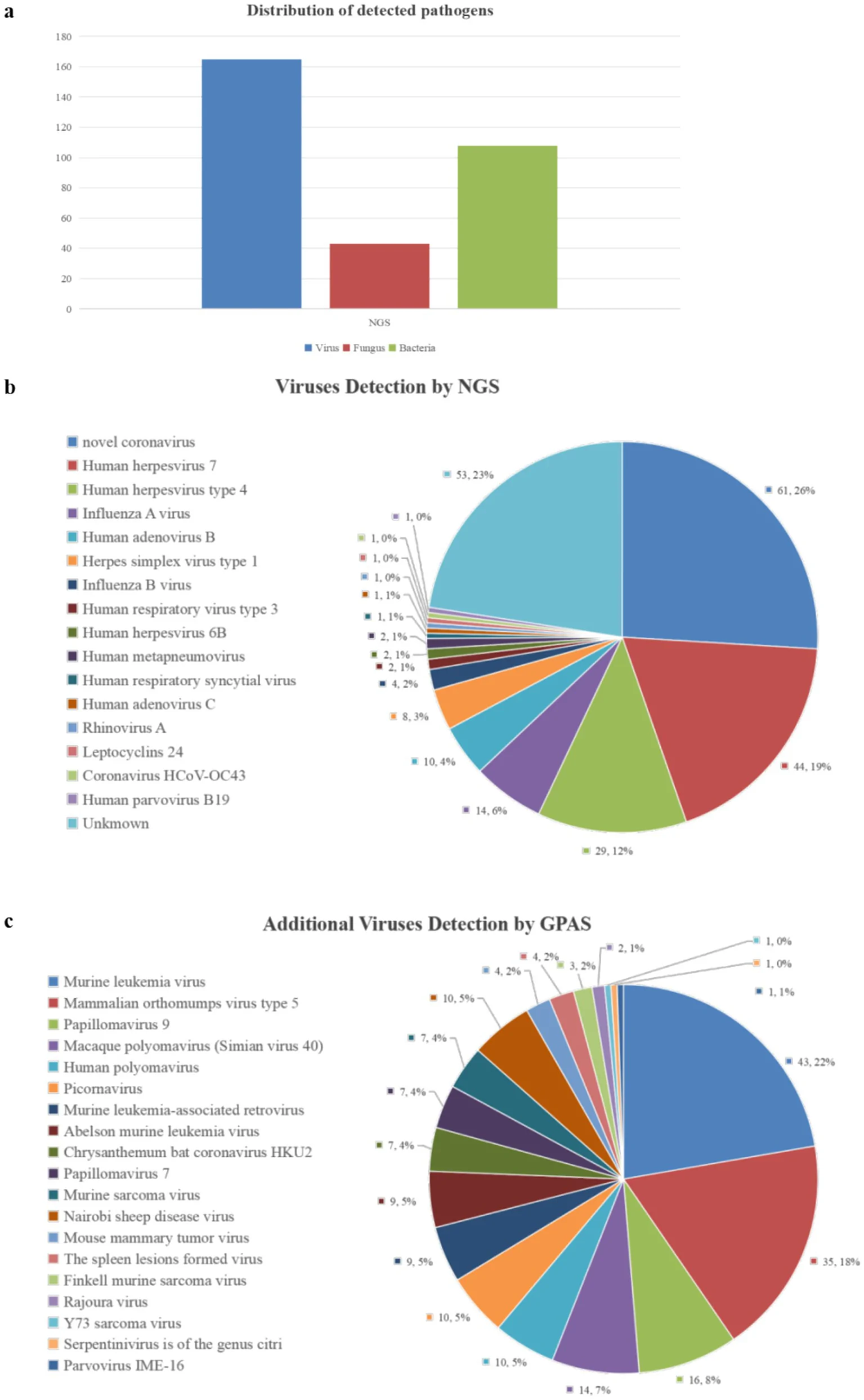
Taxonomic annotation of pathogens species. Classification and proportion of pathogens detected by different methods. **(a)** The number of cases of viral, fungal, and bacterial infection among the 218 patients; **(b)** The proportion of viruses detected by NGS; **(c)** The proportion of other viruses detected by GPAS.

In this study, the pathogenic sequences detected by mNGS in the throat swabs of these 218 patients were analyzed by GPAS again. The mNGS results revealed that bacterial infection was detected in 108 patients (49.5%), fungal infection was detected in 43 patients (19.7%), and viral infection was detected in 165 patients (75.7%) (Figure 2a). More specifically, SARS-CoV-2 was the most frequently detected virus (*N* = 61, 37%) in the mNGS analysis, followed by human herpesvirus (HHV) (Type 7: *N* = 44, 26.7%; Type 4: *N* = 29, 17.6%) and influenza A virus (*N* = 14; 8.5%) (Figures 2a,b). The in-depth analysis results based on GPAS suggested that among other viruses that were not detected by mNGS, mouse leukemia virus exhibited the largest detection ratio (*N* = 43, 23%), followed by mammalian orthorubulavirus 5 (*N* = 35, 19%) and other 19 viruses. Of note, the positive detection results of NSDV were found in 10 patients (4.6%) with the other viruses (Figure 2c). However, there have been no reports on the detection of NSDV in the respiratory tract in the Chinese population.

### 3.3 Epidemiological investigation of NSDV infection in the upper respiratory tract of patients

**Figure 3:**
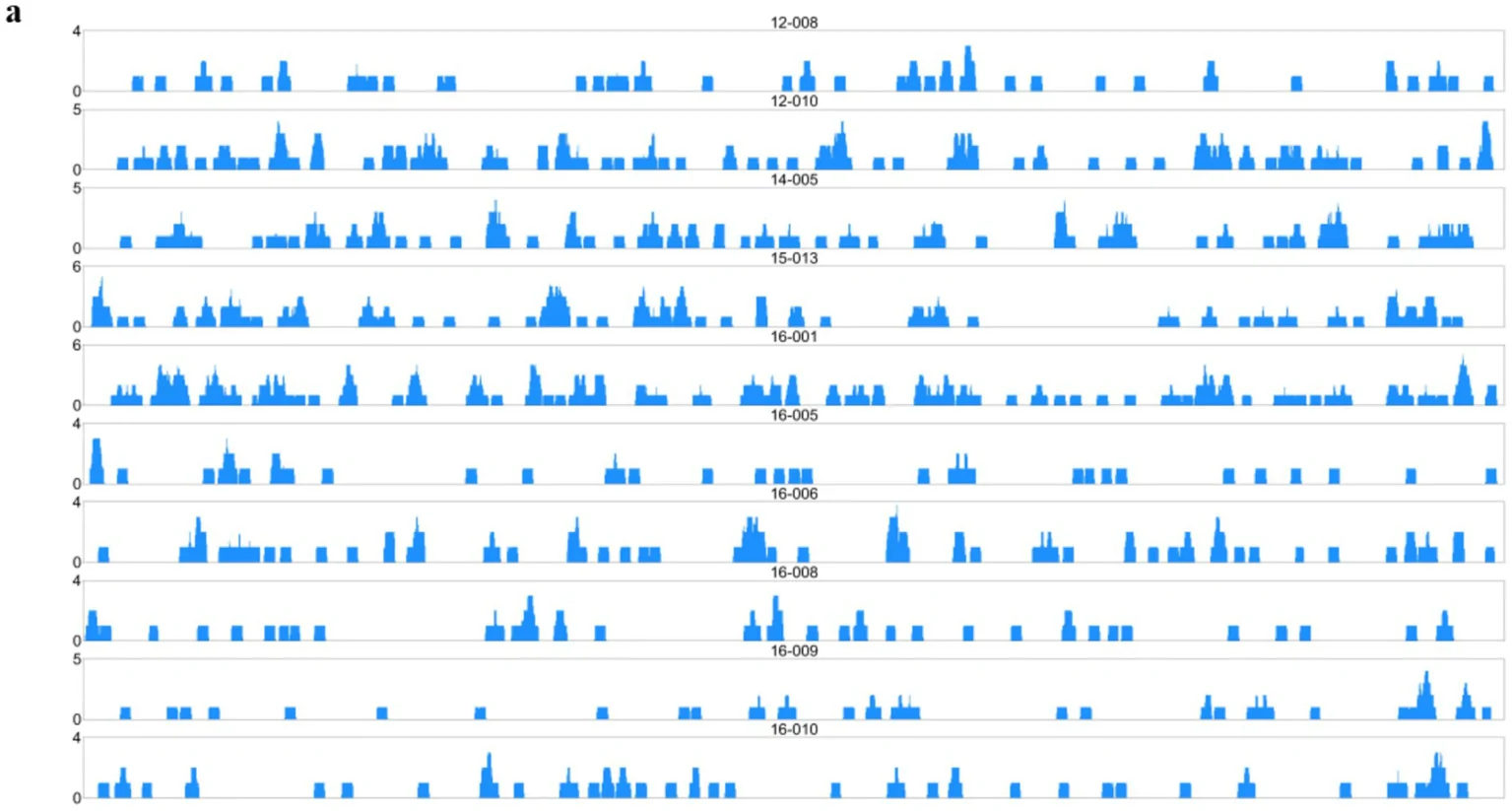
Relative abundance and correlation analysis of NSDV in patients with NSDV infection. The relative abundance of NSDV sequences in 10 patients with NSDV infection detected by GPAS.

**Figure 4:**
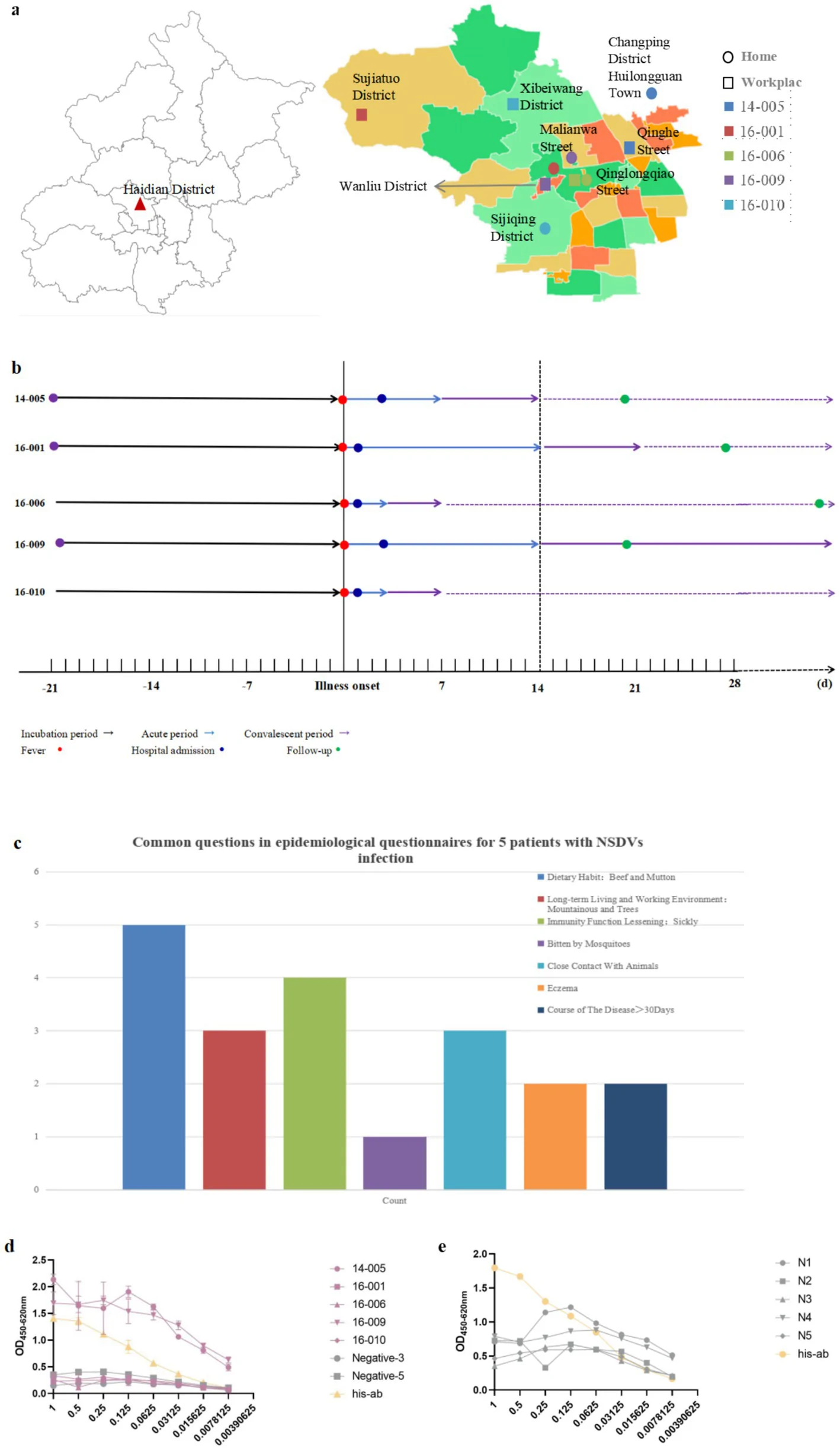
Activity trajectory and timeline of patients with NSDV infection and serological validation. **(a)** The distribution of life and work of patients with NSDV infection detected in Haidian district of Beijing (China); **(b)** The investigation timeline of 5 patients with NSDV infection from discomfort symptoms to complete recovery; **(c)** Common questions of the epidemiological questionnaire in 5 patients with NSDV infection; **(d)** The expression of serum NSDV antibodies in 5 patients with NSDV infection detected by ELISA; **(e)** The expression of serum NSDV antibodies in healthy controls detected by ELISA.

In this study, medical follow-up was performed for these 10 NSDV-positive patients detected by GPAS (Figure 3), and the clinical data of these patients for a total of 8 months (January 2025 to August 2025) before and after NSDV detection were also analyzed. The results indicated that these NSDV-positive patients were primarily manifested as coughing (70%), expectoration (70%), pharyngalgia (70%), muscle soreness (90%), and fatigue (90%). Among these patients, 7 patients (70%) had absolute lymphocyte counts below the normal range (Table 1). The informed consent of 5 patients was obtained through further telephone follow-up to collect their throat swab samples and peripheral blood samples again. Besides, a detailed investigation was conducted on these 5 patients, including name, age, gender, ethnicity, contact information, activity trajectory 3 months before infection, home address, neighborhood around home, family members, activity trajectory of family members, occupation, work address, workplace environment, commuting mode, road environment, eating habits, catering address, initial symptoms, onset time, treatment time, duration of symptoms, symptoms of re-treatment, time of re-treatment, and whether they were in contact with animals, whether they were bitten by mosquitoes, and whether they passed through forests, grasslands, parks, and zoos before infection (7–14 days). The activity trajectory of these patients and the timeline 7 weeks before and after infection were plotted (Figures 4a–c). Through further analysis, it was found that there were some similarities among these 5 patients. For instance, they had common eating habits, which were reflected as a preference for beef and mutton (*N* = 5, 100%). Besides, they shared living and working environments, which were characterized by mountainous areas and abundant vegetation (*N* = 3, 60%). In addition, they recognized decreased immunity since the infection of SARS-CoV-2 in 2022, which was proved by the increased number of illnesses (*N* = 4, 80%). Further, they reported a history of close contact with domestic pets (*N* = 3, 60%; 1 patient had contact with a pet rabbit, 1 with a hamster, and 1 with a pet pig, respectively). Moreover, 2 patients (40%) had sought medical advice due to skin rash. However, these patients did not have a defined history of tick bites. The fever of these patients was mitigated within 3 days, and coughing and expectoration lasted within 3–14 days. The discomfort symptoms of these patients completely disappeared after 7–21 days, without sequelae after recovery. Among them, 2 patients developed lung patches and ground glass images in the initial treatment, and they were treated by the intravenous infusion of antibiotics in the outpatient. For the remaining 3 patients, the symptoms were relieved after oral administration of Chinese patent medicines. In addition, 1 patient had a history of multi-regional travel within 14 days before fever (Shanxi-Shandong-Beijing) (Figure 4).

### 3.4 Serological validation of NSDV infection in the upper respiratory tract of patients

In these 5 patients who agreed to undergo the collection of throat swab samples and peripheral blood samples again, the collected peripheral blood samples were subject to serum enrichment. The His-Gn (NSDV) antigen was constructed according to the sequence of NSDV. The presence or absence of antibodies to NSDV in the serum was detected by indirect ELISA. The serum samples of 2 healthy controls were used as negative controls (H-1 and H-2), and the His-tag antigen was used as a positive control (his-positive). The experimental results showed that the serum of Patients 14-005 and 16-009 had positive detection results for NSDV antibodies (Figure 4d). Besides, the results were verified by replicate experiments (Figure 4d). In replicate experiments, the serum samples of healthy controls were added as controls, and these serum samples were fully negative for these antibodies (Figure 4e). This further confirmed that the IgG antibodies to NSDV can still be detected in the serum of patients with upper respiratory tract infection of NSDV about 3 months after infection. This finding corroborated for the first time that there was a possibility of latent infection for tick-borne NSDV in the human upper respiratory tract in Beijing (China).

### 3.5 Host associations of NSDV infection in the upper respiratory tract of patients

Besides, a cluster analysis was performed preliminarily on the pathogenic bacteria detected by mNGS in these 218 patients. The correlation between the detected pathogens and the immune status of their hosts was explored by analyzing the correlation network between omics datasets, and the findings were consistent with the statistical analysis results. A low level of lymphocytes and white blood cells and tumors (*Z* = 6.8441; *p* = 0.0089) (Table 2). It is worth analyzing that: Tumor was identified as the strongest and most statistically significant risk factor for NSDV positivity, with an odds ratio (OR) of 3.330 (95% CI: 1.352–8.199; *p* = 0.0089). This indicates that patients with a tumor had a 3.33-fold higher risk of testing NSDV-positive compared to those without a tumor. The confidence interval lies entirely above 1, supporting the robustness of this effect and its clear clinical relevance. In contrast, increasing age was associated with a reduced risk of NSDV positivity (OR = 0.949; 95% CI: 0.913–0.987; *p* = 0.0085). For each one-year increase in age, the risk of NSDV positivity decreased by approximately 5.1%, suggesting a protective effect. The confidence interval does not include 1, confirming statistical significance. Clinically, this may indicate that NSDV positivity is more prevalent among younger populations, a finding that could be attributable to the more robust immune responses and antibody production typically observed in younger patients. All *p*-values for the remaining variables exceeded 0.05, indicating no statistical significance. Although the 95% confidence intervals of their odds ratios all extended above 1, suggesting an uncertain direction of effect, the data indicate that smoking and positive imaging findings may represent potential risk factors (Table 2).

**Table 2:** Results of multivariate logistic regression analysis for NSDV positivity.

Variables	Regression coefficient	Standard error	Statistic	*P*	OR	OR (95%CI)	
						Floor	Ceiling
Age	−0.0524	0.0199	6.9215	0.0085	0.949	0.913	0.987
Sex	0.3425	0.5822	0.3462	0.5563	1.409	0.450	4.409
Smoke	0.5881	0.6561	0.8036	0.3700	1.801	0.498	6.514
Drink	−0.5139	0.6258	0.6744	0.4115	0.598	0.175	2.039
Diabetes	0.2186	0.5763	0.1439	0.7044	1.244	0.402	3.850
Tumor	1.2029	0.4598	6.8441	0.0089	3.330	1.352	8.199
Positive imaging	0.3399	0.5005	0.4612	0.4971	1.405	0.527	3.747
Lymphocyte	0.0198	0.0285	0.4834	0.4869	1.020	0.965	1.078

Goodness-of-fit test results: χ^2^ = 5.79, *p* = 0.6713.

## 4 Discussion

In this study, NSDV was found in the upper respiratory tract samples in Beijing (China), and these patients subsequently underwent serum antigen verification. The results revealed that 2 patients remained seropositive for NSDV antibodies 4 months after the detection of NSDV sequences in the upper respiratory tract. This finding further corroborated that there was a possibility of latent infection for tick-borne NSDV in the human upper respiratory tract in Beijing (China). Additionally, the correlation between NSDV and its hosts was analyzed through multi-omics approaches based on the clinical symptoms, physicochemical examination results, and epidemiological investigation results of these patients with NSDV infection. The results showed that patients infected with NSDV were primarily manifested as mild influenza-like symptoms, which may be resolved after symptomatic treatment. When patients are immunocompromised, especially under a low level of lymphocytes or the presence of tumors or underlying conditions that may affect human immunity, they may be prone to viral infection.

We characterized the epidemiological, clinical, and laboratory features of NSDV infection and further identified independent risk factors using multivariable logistic regression analysis. Despite the limited sample size of NSDV-positive cases (*n* = 10), several consistent and clinically meaningful patterns emerged. NSDV-positive patients were predominantly younger than 40 years of age, with no cases identified in individuals over 60 years. This age distribution was confirmed by multivariable analysis, which identified younger age as a significant independent protective factor. This finding suggests that NSDV infection exhibits an age-specific susceptibility pattern similar to several other viral infections, including dengue virus, chikungunya virus, and primary Epstein–Barr virus infection, where younger adults account for the majority of symptomatic cases (Guzman and Harris, 2015; Weaver and Lecuit, 2015). Possible explanations include age-dependent differences in immune competence, prior exposure to cross-reactive antigens, or behavioral exposure patterns. Notably, the absence of NSDV-positive cases in the elderly (>60 years) raises the hypothesis of pre-existing herd immunity from childhood exposure to related pathogens, analogous to the age distribution observed in the 2009 Hemagglutinin Type 1 Neuraminidase Type 1 (H1N1) influenza pandemic (Sette and Crotty, 2020).

Tumor history emerged as the strongest factors associated with NSDV positivity. Patients with a history of tumor had more than threefold higher odds of testing positive for NSDV compared to those without. This finding has important clinical implications. Tumor patients often exhibit varying degrees of immunosuppression due to the malignancy itself or secondary to anticancer therapies such as chemotherapy, radiotherapy, or targeted agents (Rolston, 2014). Immunocompromised hosts are known to be more susceptible to viral infections and may also experience prolonged viral shedding and more severe clinical courses (Shahani et al., 2017). Therefore, NSDV should be included in the differential diagnosis of febrile illness in cancer patients, particularly those who are young or have recent exposure history.

No significant associations were observed for sex, smoking, alcohol consumption, diabetes, or positive imaging findings, suggesting that these factors do not modulate susceptibility to NSDV infection. Lymphopenia was the most striking laboratory abnormality, present in 70% of GPAS-positive patients, compared with 45.4% of general fever patients and only 9.7% of healthy controls. Lymphopenia is a hallmark of many acute viral infections, including influenza, COVID-19, dengue, and Ebola, and is thought to result from direct viral cytopathic effects, lymphocyte sequestration, or cytokine-mediated apoptosis (Malavige and Ogg, 2013; Clyne and Olshaker, 1999). However, in the multivariable logistic regression model, absolute lymphocyte count was not an was not significantly associated of NSDV positivity. This apparent discrepancy warrants explanation. Lymphopenia is a non-specific acute-phase response common to many infectious and inflammatory conditions. Its association with NSDV positivity in univariable analysis likely reflects its role as a general marker of acute viral infection rather than a specific predictor of NSDV. Furthermore, lymphocyte count may be collinear with age and tumor status, as both older age and malignancy are associated with lower baseline lymphocyte counts. The small sample size may also have limited statistical power to detect a modest independent effect. Therefore, while lymphopenia is a useful screening clue, it should not be used as a standalone diagnostic criterion for NSDV. The overall profile of NSDV infection—young adult predominance, systemic symptoms, lymphopenia, and blunted CRP response—shares features with several known viral pathogens, most notably influenza virus and moderate COVID-19. However, the relatively low frequency of upper respiratory symptoms (40–50%) distinguishes NSDV from typical rhinovirus or endemic coronavirus infections (Memoli et al., 2014). Unlike dengue, thrombocytopenia and hemorrhagic signs were not reported, though platelet data were not systematically collected. Unlike SARS-CoV-2, the absence of anosmia or severe pneumonia in this small cohort precludes direct comparison.

In this study, NSDV was detected in the upper respiratory tract of humans, and the presence of serum antibodies was confirmed about 3 months after infection. However, these findings cannot prove the association between febrile diseases and NSDV. As only a few NSDV-infected patients were identified in this study, active follow-up and monitoring of these patients should be performed. In addition, the sample size needs to be expanded to further identify more patients with NSDV infection and clarify the clinical features of these patients. The serotypes of the same virus are closely related at the DNA level and often exhibit similar biological characteristics (Zahradnik et al., 1980). However, the genome-wide and homology analyses of NSDV were not performed in this study. Therefore, the phenotype of this virus is not fully elucidated, and the prevalence and pathogenicity of NSDV serotypes detected in the upper respiratory tract cannot be determined through this study. The detection of NSDV needs to be further explored from the molecular, genetic, and morphological perspectives. Moreover, the most important host defense of the human body against viral infection is to mediate cellular immunity.

Viruses have also developed a variety of immunomodulatory strategies to evade the antiviral infection mechanisms of the immune system. For instance, viruses can establish a latent period through specific transcriptional silencing mechanisms and persist in the host organism. During this period, viral gene expression is inhibited, viral particles are not produced, and continuous immune surveillance is carried out to maintain latency. When the body is immunosuppressed or in an immunocompromised state, the virus in the latent period will be reactivated through inflammation, infection, and injury, leading to the occurrence of fatal viral infection (Zahradnik et al., 1980; Forte et al., 2020; Schwartz and Stern-Ginossar, 2023). Therefore, the antagonistic ability of host antiviral innate immunity determines the severity of virus infection and the prognosis of patients (Liu et al., 2014). This antagonistic mechanism may be related to primary host factors (Chen et al., 2024), host restriction factors (Chen et al., 2022; Li et al., 2019), ncRNAs (Chang et al., 2020), metabolic pathways, and endoplasmic reticulum stress (Jiang et al., 2022). The NSDV in the upper respiratory tract detected in this study did not cause widespread transmission in the respiratory tract and serum, which may be related to its specific serophenotype and the characteristics of latent infection. This needs to be further explored in subsequent studies.

This study has several strengths, including the inclusion of a healthy control group, detailed clinical phenotyping, and multivariable adjustment to identify independent risk factors. However, limitations must be acknowledged. The small number of NSDV-positive cases (*n* = 10) limits statistical power and precludes subgroup analyses. Direct statistical comparisons between NSDV-positive and NSDV-negative fever patients were not performed for all variables. Data on viral load, disease duration, radiographic severity, treatment, and long-term outcomes were unavailable. The tumor history variable did not distinguish between active malignancy, remission, or specific treatment regimens. Finally, the single-center design may limit generalizability. Future prospective multicenter studies with larger sample sizes are needed to validate these findings, develop a clinical prediction rule, and define the natural history, transmission dynamics, and optimal management of NSDV infection.

Based on previous research results, host antiviral innate immunity may be clarified by further investigating the known antagonistic mechanism and exploring the unknown antagonistic mechanisms by referring to the research ideas of known antagonistic mechanisms among different viruses. Delving into the viral dynamic antagonism of host antiviral innate immunity, especially the dynamic interaction between viral proteins and host biomolecules, will be a great challenge for future research.

## 5 Conclusion

NSDV infection typically presents with systemic symptoms including muscular stiffness (90%) and malaise (90%), followed by lower respiratory symptoms such as cough and sputum (70% each). Upper respiratory symptoms (nasal congestion, rhinorrhea) are less prominent.

Young adults (<40 years) presenting with fever, especially those with a history of tumor, accompanied by lymphopenia (though not independently predictive, still a useful clue), and normal or mildly elevated CRP, With clinical features including muscular stiffness, malaise, cough, and sputum. For such patients, targeted virologic testing should be performed early, and empirical antibiotic therapy should be reconsidered in the absence of clear bacterial indicators.

## Data Availability

The raw data supporting the conclusions of this article will be made available by the authors, without undue reservation.

## Footnotes

1
https://sa.nanhulab.ac.cn
