# Correction: Detection and host associations of Nairobi sheep disease virus in the human upper respiratory tract in Beijing, China

**DOI:** 10.3389/fmicb.2026.1936768

**Published:** 2026-08-06

**Authors:** Liu Pengfei, Lin Chenyu, Hou Runyu, Xie Lixin

**Affiliations:** 1 College of Pulmonary and Critical Care Medicine, The 8th Medical Centre of Chinese PLA General Hospital, Beijing, China; 2 Emergency Medicine, The 8th Medical Centre of Chinese PLA General Hospital, Beijing, China

**Keywords:** fever, infection immunity, Nairobi sheep disease virus, tick-borne virus, virus-host association analysis

Author [Li Ting] was erroneously included as an author in the published article. The correct author list reads:

[Liu Pengfei^1†^, Lin Chenyu^1†^, Hou Runyu^2*^ and Xie Lixin^1*^]

The **Author contributions** statement was erroneously given as [LP: Writing – original draft, Visualization, Data curation. LC: Data curation, Methodology, Visualization, Formal analysis, Writing – review & editing, Software, Writing – original draft. LT: Investigation, Visualization, Writing – original draft, Methodology. HR: Supervision, Project administration, Investigation, Writing – review & editing, Data curation. XL: Writing – review & editing, Data curation, Project administration, Conceptualization, Resources, Funding acquisition, Supervision.].

The correct **Author contributions** statement is [LP: Writing – original draft, Visualization, Data curation. LC: Data curation, Methodology, Visualization, Formal analysis, Writing – review & editing, Software, Writing – original draft. HR: Supervision, Project administration, Investigation, Writing – review & editing, Data curation. XL: Writing – review & editing, Data curation, Project administration, Conceptualization, Resources, Funding acquisition, Supervision.].

[Throat samples immersed inactivating preservation solution were mixed with lysis buffer (Thermo Scientific, Massachusetts, United States), protease K (Yeasen, Shanghai, China), and binding buffer (Thermo Scientific, Massachusetts, United States). This mixture was subjected to mechanical disruption via a vortex mixer and beads for a duration of 30 s. Meanwhile, a positive control and a negative control were set up to monitor the whole experiment process (from the nucleic acid extraction step) of the capture-based NGS. The positive control consisted of a mixture of *Staphylococcus aureus* (103 CFU/mL) and Peripheral Blood Mononuclear Cells (PBMCs) at a concentration of 105 cells/mL, derived from healthy donors. The negative control comprised PBMCs

(105 cells/mL) obtained from healthy donors. Following this, a simultaneous extraction of Deoxyribonucleic Acid (DNA) and Ribonucleic Acid (RNA) was performed utilizing the VAMNE Magnetic Pathogen DNA/RNA Extraction Kit (Vazyme,Nanjing,China). Nucleic acid quantification was conducted employing a Qubit 3.0 fluorometer, utilizing the double-stranded DNA (dsDNA) and RNA high sensitivity assay kits. For the synthesis of cDNA and the preparation of the sequencing library, the HieffNGS^®^C37P4 OnePot cDNA & gDNA Library Prep Kit (Yeasen, Shanghai, China) was utilized, adhering to the manufacturer's instructions. Target sequence enrichment was achieved by incubating the samples with a set of 1,872 microorganism-specific probes (GenePlus, Beijing, China) for an approximate duration of 4 h, followed by an 18-cycle PCR amplification of the captured products. The probes target 1,124 bacteria, 218 fungus, 157 DNA viruses, 317 RNA viruses, and 56 parasites. The amplified products were then processed to form DNA nanoballs (DNBs). Sequencing was executed on the DNBSEQ-G99 platform with 100-bp single-end reads, targeting a sequencing depth of 5 million reads.].

A correction has been made to the section [**Section 2**, *Section 2.2*, paragraph 2]:

“[DNA was extracted from 1 mL BALF samples using a QIAamp^®^ UCP Pathogen DNA Kit (Qiagen, Valencia, CA, USA) according to the manufacturer's instructions. Human DNA was removed using Benzonase (Qiagen, Valencia, CA, USA) and Tween20 (Sigma, St. Louis, Missouri, USA). The QIAamp^®^ Viral RNA Kit (Qiagen, Valencia, CA, USA) was applied to extract total RNA, and ribosomal RNA was removed using a Ribo-Zero rRNA Removal Kit (Illumina, San Diego, CA, USA). RNA was reverse transcribed, and amplified by Ovation RNA-Seq system (NuGEN, CA, USA). Following fragmentation, the library was constructed based on combined DNA and reverse transcribed using Ovation Ultralow System V2 (NuGEN, CA, USA) and the concentration of the library was measured using Qubit. Sequencing was executed on Illumina Nextseq 550Dx with 75-bp single-end reads. For negative controls, peripheral blood mononuclear cell (PBMC) samples with 10^5^ cells/mL from healthy donors in parallel were prepared with each batch, using the same protocol, and sterile deionized water was extracted alongside the samples to serve as non-template controls (NTC). NTCs were subjected to the complete assay workflow, mirroring the procedures applied to clinical samples, commencing with nucleic acid extraction.

Raw sequencing data was processed using software Fastp to remove reads containing adapters or ambiguous “*N*” nucleotides and low-quality reads. Low-complexity reads were removed by Kcomplexity with default parameters. Human sequence data were identified and excluded by mapping to a human reference genome (hg38) using Burrows–Wheeler Aligner software. Microbial reads were then aligned to self-building database with SNAP v1.0 beta.18. Approximately 20 million reads were generated for each sample. For pathogen with background reads in negative control, a positive detection was reported for a given species or genus if the reads per million (RPM) ratio was ≥10, where the RPM ratio was defined as the RPMsample/ RPMNTC. For pathogen without background reads in negative control, the RPM threshold for a positive detection was set ≥0.05.]”

The original version of this article has been updated.

